# *Clausena lansium* (Lour.) Skeels Leaf Volatile Oil Emulsion Alleviates Heat Stress-Induced Liver Injury in Broilers Accompanied by Modulation of Autophagy- and Ferroptosis-Related Pathways

**DOI:** 10.3390/ani16193045

**Published:** 2026-09-28

**Authors:** Hui-Lin Li, Yang Yang, Jia-Ci Cai, Yu-Ting Zhang, Shun Tian, Yong-Ming He, Lu-Ping Tang

**Affiliations:** School of Animal Science and Technology, Foshan University, Foshan 528225, China; silencelin0917@163.com (H.-L.L.); yangmiemie14760@gmail.com (Y.Y.); sanyecai18@163.com (J.-C.C.); ccoise1207@outlook.com (Y.-T.Z.); qm111011@163.com (S.T.); ymhe@fosu.edu.cn (Y.-M.H.)

**Keywords:** heat stress, broiler, liver injury, autophagy, ferroptosis

## Abstract

Heat stress (HS) severely compromises broilers’ health and production performance. This study aimed to evaluate the protective effects of *Clausena lansium* (Lour.) Skeels (wampee) leaves volatile oil emulsion (WVOE) against HS-induced liver injury in broilers and to investigate its underlying mechanisms. A total of 120 broilers were randomly allocated into five groups (3 replicate cages per group, 8 broilers per cage): Control (22 ± 2 °C), HS (Heat stress, 35 ± 2 °C and 80 ± 10% relative humidity for 8 h/day for 7 consecutive days), VitC (HS + Vitamin C, 60 mg/kg), WVOE-H (HS + WVOE, 3 mg/kg), and WVOE-L (HS + WVOE, 0.75 mg/kg). VitC and WVOE were administered intragastrically 1 h after daily HS exposure. HS impaired growth performance, as evidenced by decreased final body weight and average daily gain (ADG) and an increased feed conversion ratio (FCR). WVOE-L administration reduced FCR compared with the HS group. HS also impaired liver function and induced oxidative injury, as reflected by increased serum aspartate aminotransferase (AST) and alanine aminotransferase (ALT) activity, decreased total protein (TP) and albumin (ALB) levels, elevated hepatic malondialdehyde (MDA) content, reduced superoxide dismutase (SOD) activity and glutathione (GSH) levels. WVOE and VitC ameliorated these alterations, thereby alleviating liver injury and oxidative stress. Both WVOE and VitC treatment partially ameliorated these alterations and were associated with milder histopathological changes, suggesting alleviation of HS-induced liver injury and oxidative stress. Under HS conditions, WVOE decreased HSP70 and IL-6 protein expression and was accompanied by changes in autophagy-related signaling, including increased LC3-II/LC3-I ratio, protein expressions of Beclin-1, ULK1, ATG4B, ATG5, and ATG7, and reduced p62 accumulation. WVOE increased gene and protein expression of GPX4 and SLC7A11 and decreased ACSL4 expression. In conclusion, WVOE alleviated several indices of HS-induced liver injury in broilers, accompanied by changes in autophagy-related signaling and ferroptosis-related molecular responses.

## Simple Summary

Sustained high temperatures in rearing environments impair broiler health, reduce farming profits, and induce liver injury in broilers. This study evaluated the hepatoprotective effects of volatile oil emulsion extracted from *Clausena lansium* (Lour.) Skeels (wampee) leaves, a processed product prepared from essential oil isolated from wampee leaves, against heat stress-triggered liver damage in broilers. Continuous high temperature retards broiler growth, causes hepatic inflammation and oxidative injury, and disturbs cellular homeostasis. Supplementation with WVOE alleviated these adverse changes and was associated with modulation of autophagy- and ferroptosis-related pathways. These findings suggest that WVOE may serve as a potential plant-derived intervention for alleviating heat stress-induced liver injury in broilers, although its practical application requires further investigation.

## 1. Introduction

With the acceleration of global warming, heat stress (HS) has emerged as a major environmental stressor that severely compromises poultry health and production [1]. Due to the absence of sweat glands and limited capacity for heat dissipation, poultry are particularly vulnerable to high-temperature environments [2]. Sustained or excessive heat exposure disrupts physiological homeostasis, impairs immune defense, and ultimately impairs growth performance [3]. Furthermore, HS predisposes poultry to infection diseases by impairing host resistance, which may raise serious food safety concerns and economic losses in the poultry industry [4].

The liver, a crucial organ responsible for metabolism, detoxification, and immune regulation, is highly susceptible to HS-induced injury. Veterinary and experimental studies have demonstrated that exposure to high temperatures promotes hepatic oxidative stress and mitochondrial dysfunction [5]. These pathological alterations impair the structural and functional integrity of hepatocytes, disrupt hepatic metabolic homeostasis, and ultimately contribute to impaired growth and reduced productive performance in poultry [6]. In response to oxidative stress, autophagy is generally activated as an essential cellular defense mechanism to repair or eliminate damaged proteins and organelles, particularly dysfunctional mitochondria, thereby maintaining cellular homeostasis [7]. However, under HS, autophagy-related signaling may be disrupted or inappropriately down-regulated, leading to inadequate clearance of damaged organelles and further aggravation of hepatic injury [8].

In traditional Chinese medicine, *Clausena lansium* (Lour.) Skeels, commonly known as Wampee, is a Rutaceae plant with a long history of medicinal application. Wampee leaves are one of its medicinal parts and have traditionally been used for clearing heat, resolving phlegm, and relieving inflammation-related symptoms [9,10]. Modern studies indicate that the pharmacological effects of wampee leaves are closely associated with the content and constituents of their volatile oils [9]. However, studies on wampee leaf volatile oil remain limited, with most available research focusing on chemical composition characterization, whereas its pharmacological activities, bioactive material basis, and potential application in poultry remain insufficiently understood [11].

Our previous study demonstrated that steam distillation was the optimal method for extracting wampee leaf volatile oil, with an oil yield of 0.50% after process optimization. Gas chromatography–mass spectrometry (GC–MS) analysis characterized its chemical profile and showed that the volatile oil was mainly composed of aromatic hydrocarbons, terpenoids, and their oxygenated derivatives, including β-ocimene, α-bisabolol, and palmitic acid [12]. These bioactive components have been reported to possess antioxidant, anti-inflammatory, antimicrobial, and hepatoprotective activities [13]. Furthermore, the volatile oil was formulated as an oil-in-water emulsion, termed wampee leaf volatile oil emulsion (WVOE), to improve its physicochemical stability and ease of administration compared with the crude volatile oil [14]. Plant essential oils and phytogenic bioactive compounds can alleviate heat stress-related damage by improving antioxidant capacity and reducing inflammatory or oxidative injury. Previous studies have mainly focused on the extraction optimization, and chemical characterization of wampee leaf volatile oil. However, the pharmacological activities of WVOE on HS-induced liver injury in broilers remains unclear. In the present study, a broiler model of HS was established to evaluate the protective effects of WVOE on HS-induced liver damage and its underlying molecular mechanisms. The results are expected to provide theoretical basis for the development of WVOE as a natural intervention for mitigating heat stress-related liver injury in poultry production.

## 2. Materials and Methods

### 2.1. Preparation of WVOE

The leaves of *Clausena lansium* (Lour.) Skeels were collected from Yunan County, Yunfu City, Guangdong Province, China, and were authenticated as leaves of the seedless wampee tree. The dried leaf powder sample (No. 2022081601) was deposited at the veterinary drug research laboratory, Foshan University, China, for future reference. The WVOE used in the broiler experiment was prepared from volatile oil freshly extracted from the same batch of dried wampee leaf powder used in our previous GC–MS analysis. The extracted volatile oil was immediately formulated into WVOE and stored at 4 °C until use. Leaves were dried indoors at room temperature, pulverized into fine powder, and passed through a 100-mesh sieve before further processing. Twenty grams of wampee leaf powder were placed into a 500 mL round-bottom flask, followed by the addition of 200 mL of ultrapure water (material-to-liquid ratio of 1:10). After soaking for 2 h, the essential oil was extracted by hydrodistillation for 4 h. After hydrodistillation, the distillate was extracted three times with ethyl acetate, and the upper ethyl acetate phase was collected. The combined ethyl acetate extracts were dried over anhydrous sodium sulfate for 10 h and then filtered. Ethyl acetate was subsequently evaporated in a water bath to obtain the wampee leaf volatile oil. The WVOE was prepared as an oil-in-water (O/W) emulsion with an oil phase-to-aqueous phase ratio of 1:9 (*v*/*v*), in which the oil phase consisted of wampee leaf volatile oil and Tween 80 as an emulsifier at a ratio of 1:1 (*v*/*v*). Briefly, 100 mg of wampee leaf volatile oil was thoroughly mixed with 100 μL of Tween 80 to form the oil phase. The oil phase was then added dropwise into 1800 μL of water at 80 °C under magnetic stirring at 500 rpm. The mixture was then stirred for an additional 30 min to obtain WVOE with a final wampee leaf volatile oil concentration of 50 mg/mL [14].

To provide quantitative information on the major chemical constituents of the preparation, 37 compounds were identified in wampee leaf volatile oil by gas chromatography–mass spectrometry (GC–MS) in our previous study [12]. These compounds were mainly classified as aromatic hydrocarbons, terpenoids, and their oxygenated derivatives. The major constituents of wampee leaf volatile oil included 5,5-dimethyl-1-vinylbicyclohexane (13.0509%), palmitic acid (13.8385%), β-ocimene (6.5805%), phytol (5.7725%), caryophyllene oxide (3.0777%), α-bisabolol (2.8441%), and spathulenol (1.7751%).

### 2.2. Animals and Experimental Design

A total of 120 healthy yellow-feathered broilers (approximately equal number of males and females) were provided by Xinguang Agricultural and Animal Husbandry Co., Ltd. (Foshan, China). All 1-day-old broilers in an environmentally controlled chamber for 13 day, with the temperature and relative humidity maintained at 22 ± 2 °C and 60 ± 10%, respectively, under a 12 h light/dark cycle. Broilers were housed in cages (100 cm × 60 cm × 70 cm, each cage represented one replicate and was equipped with a feeder and a drinker), with eight birds per cage and ad libitum access to feed and water. After acclimation, mixed-sex broilers were weighed individually at 14 days of age and randomly assigned to treatments and replicates after body weight balancing, with 24 birds per group and 3 replicates of 8 birds each: Control, HS (Heat Stress, 35 ± 2 °C and 80 ± 10% humidity for 8 h/day for 7 consecutive days), VitC (HS + Vitamin C, 60 mg/kg), WVOE-H (HS + WVOE-H, 3 mg/kg), and WVOE-L (HS + WVOE-L, 0.75 mg/kg) group. The WVOE dose was calculated based on the volatile oil content. The WVOE stock emulsion was diluted daily with phosphate-buffered saline (PBS) according to body weight, and each bird received 1 mL by oral gavage 1 h after daily HS exposure. Birds in the control and HS groups received an equal volume of sterile PBS. The HS conditions and vitamin C dose were selected based on a previous heat stressed-broiler study [6]. The dose of WVOE-L (0.75 mg/kg) was derived from the effective dose used in our previous mouse antibacterial study through interspecies dose conversion [15], while a higher dose of WVOE-H (3 mg/kg) was included to asses dose-related effects. The broilers were feed a commercial diet (product code: 510; Shijiazhuang, China) formulated for 1–21-day-old broilers according to the product standard Q/01ZD001-2024 [16]. The available ingredient information and nutrient composition based on the product label and manufacturer’s inspection report are presented in Table 1.

For the heat stress protocols, broilers (14 d of age) in the HS and drug treatment groups were subjected to 35 ± 2 °C and 80% ± 10% relative humidity for 8 h/day (08:00 to 16:00) for 7 consecutive days, while maintained at the same conditions as the control group (22 ± 2 °C) for the rest of the day. HS exposure was conducted in three temperature-controlled incubators. One replicate cage from each HS-related group (HS, VitC, WVOE-H, and WVOE-L) was placed in each incubator. The replicate cages were rotated daily among the three incubators and between the upper and lower levels to minimize potential incubator- and position-related effects. The Control group was maintained at 22 ± 2 °C in the room outside the heat-stress incubators throughout the experimental period. During HS exposure, Temperature and relative humidity were recorded every 30 min, with the temperature maintained at 35 ± 2 °C. Broilers in the drug treatment groups received VitC or WVOE by oral gavage. At 21 days of age, all broilers were euthanized by CO_2_ inhalation, and liver tissues were collected for subsequent analyses. All animals procedures were conducted in accordance with the guidelines for the care and use of experimental animals established by the Ministry of Science and Technology of the People’s Republic of China (Approval number: 2006-398) [17] and was approved by the experimental animal ethics committee of Foshan University (FOSU1951250723). All efforts were made to minimize animal suffering.

**Table 1:** Main ingredient composition and nutrient levels of the basal diet.

Item	1–21 d
Main ingredient composition	Corn, soybean meal, corn gluten meal, soybean oil, dicalcium phosphate, limestone powder, sodium chloride, amino acids, vitamin and mineral premix, and enzyme preparation
Moisture, %	10.9
Crude protein, %	21.0
Crude fat, %	5.9
Crude fiber, %	3.1
Crude ash, %	5.2
Calcium, %	0.60–1.20
Total phosphorus, %	≥0.50
Sodium chloride, %	0.20–0.80
Methionine, %	0.40–0.90

Note: The main ingredient information and guaranteed nutrient levels were obtained. The analyzed values for moisture, crude protein, crude fat, crude fiber, and crude ash were obtained from the manufacturer’s inspection report. The allowable deviations for feed analysis results were judged according to GB/T 18823 [18].

### 2.3. Growth Performance Analysis

The initial body weight, final body weight, body weight gain, and feed intake were measured and calculated. To evaluate the growth performance, the body weight of each broiler was recorded from 14 to 21 d of age. Initial body weight and final body weight, individual values within each cage were averaged, and cage means were used as the experimental units for cage-level analysis (*n* = 3 replicate cages per group, 8 broilers per cage). The parameters assessed included average daily gain (ADG), average daily feed intake (ADFI), and feed conversion ratio (FCR). FCR was calculated as the ratio of ADFI to ADG (FCR = ADFI/ADG). ADG, ADFI, and FCR were calculated and analyzed at the cage level, with each cage used as the experimental unit (*n* = 3 replicate cages per group, 8 broilers per cage).

### 2.4. Sample Collection

During the experiment period, broilers were weighed after fasting. After the final HS exposure, blood samples were collected from the jugular vein for the determination of serum biochemical indicators related to liver function. The broilers were then euthanized in strict accordance with the Laboratory animal-Guidelines for the euthanasia (GB/T 39760-2021) [19]. Liver tissues were collected, weighed, and recorded. A portion of the liver tissue was fixed in 4% paraformaldehyde for histopathological analysis, while the remaining samples were snap-frozen in liquid nitrogen and stored at −80 °C for subsequent gene and protein expression analysis [20].

### 2.5. Serum Biochemical Analysis

At 21 days of age, 1 mL of jugular venous blood was collected into anticoagulant-free vacuum blood collection tubes. The blood samples were allowed to clot at room temperature for 2 h, and then centrifuged at 3000 rpm for 10 min at 4 °C. The serum supernatant was carefully transferred into 1.5 mL sterile EP tubes and stored at −20 °C [21]. Serum biochemical indices, including aspartate aminotransferase (AST, 105-000443-00), alanine aminotransferase (ALT, 105-000442-00), total bilirubin (T-Bil, 105-000454-00), direct bilirubin (D-Bil, 105-000455-00), total protein (TP, 105-015578-00), albumin (ALB, 105-000450-00), and lactate dehydrogenase (LDH, 105-000446-00), were measured using a Mindray automatic biochemical analyzer (Mindray, Shenzhen, China). Serum biochemical parameters were analyzed in all 24 broilers per group. For cage-level analysis, individual values within each cage were averaged, and cage means were used as the experimental units (*n* = 3 replicate cages per group, 8 broilers per cage).

### 2.6. Histological Analysis of Liver

Liver tissue samples (approximately 1 cm × 1 cm × 0.5 cm) were collected from the same region near the gallbladder of the right lobe. The samples were fixed in 4% formaldehyde, dehydrated, embedded in paraffin, and sectioned. The sections were stained with hematoxylin and eosin (H&E). Histopathological changes in liver tissues were observed under a light microscope (Bio-Rad, Hercules, CA, USA) [22]. For each animal, 5–10 fields from at least 4 sections were evaluated, with at least 3 animals in each group.

### 2.7. Hepatic Oxidation State Analysis

Hepatic glutathione (GSH), superoxide dismutase (SOD), and malondialdehyde (MDA) levels were determined using commercial assay kits obtained from Jiancheng Bioengineering Institute Co., Ltd. (Nanjing, China). The catalog numbers were as follows: GSH, Cat. No. A006-2-1; SOD, Cat. No. A001-3-2; and MDA, Cat. No. A003-1-2. Briefly, 0.1 g of liver tissue was weighed, minced, and homogenized in 1 mL of pre-cooled physiological saline using a low-temperature tissue grinder with steel beads to prepare a 10% (*w*/*v*) tissue homogenate. The homogenate was centrifuged at 3500 rpm for 10 min at 4 °C, and the supernatant was collected for analysis. GSH, SOD and MDA levels were determined using commercial assay kits according to the manufacturer’s instructions. GSH content was measured using the reaction between reduced glutathione and 5,5′-dithiobis-(2-nitrobenzoic acid), and expressed as μmol/g protein. SOD activity was determined based on inhibition of superoxide anion-mediated color development and expressed as U/mg protein. MDA content was measured using the thiobarbituric acid-reactive substances method and expressed as nmol/mg protein [23]. Liver samples from 12 broilers per group (four broilers per replicate cage) were analyzed. For cage-level analysis, individual values within each cage were averaged, and cage means were used as the experimental units (*n* = 3 replicate cages per group).

### 2.8. Western Blot Analysis

For Western blot analysis, three independent liver samples per group were analyzed, with one broiler selected from each replicate cage. Each liver sample represented one replicate cage and was considered an experimental unit. Total proteins were extracted from liver tissues using RIPA lysis buffer, and the protein concentrations were quantified using a BCA protein assay kit. Equal amounts of denatured protein (about 30 μg/lane) were separated by SDS-PAGE using 8%, 10%, or 12% gels according to target protein molecular weight. After electrophoresis, proteins were transferred onto polyvinylidene difluoride (PVDF) membranes by wet transfer system at approximately 300 mA for 30–45 min. The membranes were blocked with 5% skim milk or bovine serum albumin (BSA) at room temperature for 1 h, the membranes were incubated with specific primary antibodies overnight at 4 °C. After washing three times with TBST, the membranes were incubated with horseradish peroxidase (HRP)-conjugated goat anti-rabbit secondary antibodies for 1 h at room temperature. Finally, the protein bands were detected using an enhanced chemiluminescence (ECL) substrate (Cat. #170-5051, Bio-Rad, Hercules, CA, USA), imaged with an automated chemiluminescence imaging system (Tanon, Shanghai, China), and quantified using ImageJ software (version 1.51j8, National Institutes of Health, Bethesda, MD, USA) [6]. Phosphorylated protein levels were normalized to their corresponding total protein levels, including p-ULK1/ULK1 and p-mTOR/mTOR, whereas other target proteins were normalized to β-actin or GAPDH.

Primary antibodies against p62 (rabbit, Cat. No. 39749, 1:1000), LC3I/II (rabbit, Cat. No. 4108, 1:1000), Beclin-1 (rabbit, Cat. No. 3738, 1:1000), ULK1 (rabbit, Cat. No. 8054, 1:1000), phospho-ULK1 (Ser757) (rabbit, Cat. No. 14202, 1:1000), ATG7 (rabbit, Cat. No. 8558, 1:1000), ATG5 (rabbit, Cat. No. 12994, 1:1000), ATG4B (rabbit, Cat. No. 5299, 1:1000), mTOR (rabbit, Cat. No. 2983, 1:1000), phospho-mTOR (Ser2448) (rabbit, Cat. No. 5536, 1:1000), HSP70 (rabbit, Cat. No. 4872, 1:1000), IL-1β (rabbit, Cat. No. 12703, 1:1000), IL-6 (rabbit, Cat. No. 12912, 1:1000), β-actin (rabbit, Cat. No. 4970, 1:1000), SLC7A11 (rabbit, Cat. No. 28001, 1:1000), GPX4 (rabbit, Cat. No. 59735, 1:1000), and GAPDH (rabbit, Cat. No. 5174, 1:1000) were purchased from Cell Signaling Technology (Danvers, MA, USA). The primary antibody against ACSL4 (rabbit, Cat. No. T510198, 1:5000) was purchased from Abmart Biomedical Co., Ltd. (Shanghai, China). HRP-conjugated rabbit IgG (Cat. #SA00001-2) was purchased from Proteintech (Wuhan, China).

### 2.9. Quantitative Real-Time Polymerase Chain Reaction Assay

Total RNA was extracted from liver tissues using an RNA extraction kit (Fastagen, Shanghai, China). The purity of RNA was assessed based on the A260/A280 ratio using a micro UV-Vis spectrophotometer (Thermo Fisher Scientific, Wilmington, DE, USA). cDNA was synthesized from total RNA using HiScript IV All-in-One Ultra RT SuperMix for qPCR (Vazyme, Nanjing, China). The Quantitative real-time PCR was conducted using Taq Pro Universal SYBR qPCR Master Mix (Vazyme, Nanjing, China), together with cDNA templates and gene-specific primers. Gene-specific primers were designed by Sangon Biotech (Shanghai, China), and the primer sequences are listed in Table 2.

qRT-PCR was performed using cDNA and SYBR Green qPCR master mix on a qTOWER^3^ G real-time PCR system (Analytik Jena, Jena, Germany). The cycling conditions: initial denaturation at 95 °C for 30 s, followed by 40 cycles of denaturation at 95 °C for 10 s and annealing/extension at 60 °C for 30 s. Fluorescence signals were collected at the end of each cycle. Each sample was analyzed in triplicate, and no-template controls were included for each primer pair. Data were analyzed using qPCRsoft software (version 4.1, Analytik Jena, Jena, Germany). Relative gene expression was calculated using the 2^−ΔΔCt^ method with β-actin as the reference gene [24]. Six biological replicates per group were analyzed, with two broilers selected from each replicate cage. For cage-level analysis, individual values within each cage were averaged, and cage means were used as the experimental units (*n* = 3 replicate cages per group). Each sample was measured in technical triplicate.

### 2.10. Statistical Analysis

Data are presented as the means ± standard deviation (mean ± SD). All statistical analyses were performed using GraphPad Prism software (version 9.0.0). Continuous variables were first tested for normality using the Shapiro–Wilk test and for homogeneity of variance using the Brown–Forsythe test. For data that met the assumptions of normal distribution and homogeneity of variance, differences among groups were analyzed using one-way ANOVA followed by Tukey’s multiple comparisons test. For data with unequal variances, Welch’s ANOVA followed by Dunnett’s T3 multiple comparisons test was used. For non-normally distributed data, the Kruskal–Wallis test was used as the non-parametric omnibus test, followed by Dunn’s multiple comparisons test with Bonferroni adjustment. Multiple comparisons were performed among all treatment groups. For growth-performance parameters, cage means were used as the experimental units (*n* = 3 replicate cages per group), and the corresponding Tukey-adjusted *p*-values were used for pairwise comparisons. A *p*-value < 0.05 was considered statistically significant. Different lowercase letters indicate significant differences among groups (*p* < 0.05).

## 3. Results

### 3.1. WVOE Enhanced the Growth Performance of Broilers Under Heat Stress

The effects of WVOE on growth performance in HS-exposed broilers are presented in Table 3. Initial body weight did not differ significantly among groups. Compared with the control group, HS significantly reduced final body weight and ADG and increased FCR (*p* < 0.05), indicating impaired growth performance. Compared with the HS group, VitC significantly improved final body weight and ADG (*p* < 0.05), whereas WVOE-L significantly reduced FCR (adjusted *p* = 0.0418). These results suggest that WVOE-L improved feed efficiency in HS-exposed broilers.

### 3.2. WVOE Alleviated Heat Stress-Induced Liver Dysfunction in Broilers

As shown in Table 4, compared with the control group, HS significantly increased serum ALT, AST, and LDH activities (*p* < 0.05), whereas TP and ALB levels were significantly decreased (*p* < 0.05). Compared with the HS group, VitC and both WVOE treatments reduced serum LDH and AST activities and increased ALB levels (*p* < 0.05), while VitC and WVOE-L also reduced ALT activity (*p* < 0.05). VitC also increased the TP level compared with the HS group (*p* < 0.05). These results suggest that heat stress induced hepatocellular injury in broilers, while WVOE administration ameliorated HS-induced liver dysfunction.

### 3.3. WVOE Alleviated Liver Pathological Changes and Redox Status of Broilers Under Heat Stress

As shown in Figure 1A, gross examination revealed that the livers of broilers in HS group appeared slightly yellowish, with scattered petechial hemorrhages, accompanied by pericardial effusion. In contrast, the livers from the control and treatment groups exhibited relatively normal gross morphology, characterized by a reddish-brown color, homogeneous texture, smooth and glossy surface, well-defined margins, and soft texture, without visible pathological alterations, such as hemorrhage, swelling, or necrosis.

Histopathological changes in the liver were evaluated based on H&E-stained sections. The liver tissues in the control group displayed intact histological architecture, with regularly arranged hepatic cords and normal hepatocyte morphology. No obvious inflammatory cell infiltration, hepatocyte necrosis, hemorrhage, or other pathological alterations were observed. In the HS group, histological observations showed hepatic structural disruption, inflammatory cell infiltration, mainly neutrophils and lymphocytes, erythrocyte extravasation, hemorrhagic foci, and areas of coagulative necrosis. Compared with the HS group, the VitC and WVOE-treated groups showed relatively milder histological alterations, as evidenced by more orderly hepatocyte arrangement, better preserved structural integrity, and less apparent inflammatory responses in the portal areas. These observations suggest that WVOE may alleviate HS-induced histological liver alterations in broilers.

The hepatic redox status is shown in Figure 1D–F. Compared with the control group, HS significantly increased MDA content (*p* < 0.05), while markedly decreasing SOD activity and GSH levels (*p* < 0.05). Compared with the HS group, VitC and both WVOE treatments significantly decreased MDA content and increased GSH levels (*p* < 0.05), while both WVOE treatments also significantly increased SOD activity (*p* < 0.05). These results suggest that WVOE alleviated HS-induced oxidative liver injury by suppressing lipid peroxidation and partially restoring antioxidant capacity.

### 3.4. WVOE Modulated Autophagy-Related Protein Expression in Broilers Under Heat Stress

Autophagy is an essential mechanism for maintaining cellular homeostasis. As shown in Figure 2, HS significantly increased the p-mTOR/mTOR ratio (*p* < 0.05), suggesting alterations in autophagy initiation-related signaling. HS also decreased the levels of LC3II/I ratio, ULK1, Beclin-1, ATG7, ATG5, and ATG4B, while increasing the accumulation of p62 (*p* < 0.05). These changes suggest that HS impair autophagy-related processes, particularly autophagy initiation and autophagosome formation. Compared with the HS group, VitC and WVOE partially restored autophagy-related protein expression. Specifically, WVOE-H significantly increased Beclin-1, ATG7, ATG4B, and ATG5 levels and reduced the p-ULK1/ULK1 ratio, whereas WVOE-L significantly increased the LC3-II/LC3-I ratio, Beclin-1, ATG7, and ATG5 levels and reduced p62 accumulation and the p-ULK1/ULK1 ratio (*p* < 0.05). These results suggest that WVOE modulated autophagy-related signaling under HS conditions.

### 3.5. WVOE Modulated the mRNA Expression of Autophagy-Related Genes in Broilers Under Heat Stress

As shown in Figure 3, HS significantly decreased the mRNA levels of LC3A, LC3B, ULK1 and Beclin-1 (*p* < 0.05), whereas the mRNA levels of mTOR and p62 were increased (*p* < 0.05). Compared with the HS group, both WVOE treatments significantly alleviated these changes (*p* < 0.05). VitC showed similar effects on LC3B, ULK1, Beclin-1, mTOR, and p62 (*p* < 0.05). These results suggest that WVOE modulated autophagy-related gene expression in the liver of broilers exposed to heat stress.

### 3.6. WVOE Modulated the mRNA and Protein Expression of Ferroptosis-Related Markers in Broilers Under Heat Stress

As shown in Figure 4, HS decreased SLC7A11 and GPX4 protein expression, while increasing ACSL4 protein expression (*p* < 0.05). Together with the increased MDA content and decreased GSH level described above, these results suggest that HS promoted hepatic lipid peroxidation and was accompanied by ferroptosis-related molecular changes. WVOE-L and VitC upregulated SLC7A11 and GPX4 protein expression and downregulated ACSL4 protein expression (*p* < 0.05). Meanwhile, WVOE-H increased GPX4 protein expression compared with the HS group (*p* < 0.05). These changes suggest that WVOE alleviated HS-induced alterations in ferroptosis-related markers, accompanied by improved lipid peroxidation defense.

In addition, HS significantly upregulated ACSL4 mRNA expression and downregulated SLC7A11 and GPX4 mRNA expression (*p* < 0.05). Compared with the HS group, both WVOE treatments significantly alleviated these changes (*p* < 0.05). VitC significantly increased GPX4 and decreased ACSL4 mRNA expression (*p* < 0.05). These results indicate that WVOE modulated ferroptosis-related gene expression under HS conditions.

### 3.7. WVOE Modulated the mRNA and Protein Expression of Inflammation-Related Markers in Broilers Under Heat Stress

HSP70 is a key stress-responsive protein involved in cellular protection against HS-induced damage, whereas IL-1β and IL-6 are important pro-inflammatory cytokines. As shown in Figure 5, HS significantly increased HSP70, IL-1β, and IL-6 protein expression and upregulated HSP70 and IL-1β mRNA levels (*p* < 0.05). Compared with the HS group, VitC and both WVOE treatments significantly decreased HSP70 and IL-1β mRNA expression (*p* < 0.05). All treatments significantly reduced HSP70 protein expression, while VitC reduced IL-1β protein expression, and VitC and WVOE-L reduced IL-6 protein expression (*p* < 0.05). These findings suggest that WVOE attenuated HS-induced stress and inflammation responses.

## 4. Discussion

Heat stress (HS) is a severe environmental stressor that disrupts physiological homeostasis, and compromises growth performance and production efficiency in poultry [25]. In this study, HS impaired broiler growth performance, as indicated by reduced final body weight and ADG and an increased FCR, while ADFI showed only a numerical decreasing trend. This finding is consistent with our previous findings [26]. VitC supplementation significantly increased final body weight and ADG under HS conditions, whereas WVOE-L reduced FCR. These findings suggest that WVOE-L improves feed efficiency in broilers under heat stress. As a pivotal metabolic organ in poultry, the liver is particularly vulnerable to HS-induced injury [27]. HS can impair liver structure and function through multiple mechanisms, including oxidative stress, inflammatory activation, and metabolic disturbance [28]. Consistently, in the present study, HS induced hepatic injury, as evidenced by elevated serum AST, ALT and LDH activities, reduced TP and ALB levels, increased hepatic MDA content, decreased SOD activity and GSH levels, and induced histopathological alterations, including inflammatory cell infiltration and lymphocyte accumulation in liver tissues. Notably, treatment with WVOE and VitC partially mitigated these adverse changes, as reflected by reduced hepatic MDA content, increased SOD activity and GSH levels, and comparatively milder histological changes. These results indicate that WVOE mitigates HS-induced liver injury in broilers, although the underlying mechanisms require further elucidated.

Autophagy activation has been reported to alleviate HS-induced oxidative and maintain tissue homeostasis [29]. Beclin-1 is a key regulator of autophagy initiation and promotes autophagosome formation by coordination with autophagy-related proteins. A decreased LC3-II/LC3-I ratio accompanied by p62 accumulation generally indicate reduced autophagy-related activity or altered autophagy-related protein turnover [30]. Previous studies have shown that HS induces oxidative stress and disrupts hepatic autophagic regulation, thereby aggravating tissue damage [26]. This study found that HS markedly altered hepatic autophagy-related markers, as evidenced by a reduced LC3-II/LC3-I ratio, decreased LC3A, LC3B, Beclin-1 and ULK1 expression, p62 accumulation, and disruption of mTOR/ULK1-related signaling. These changes are consistent with impaired autophagy initiation and autophagosome formation, together with altered autophagy-related protein turnover. VitC and WVOE partially reversed these alterations. WVOE and VitC partially reversed these alterations. In particular, WVOE increased the LC3-II/LC3-I ratio and the expression of Beclin-1, ATG7, ATG5, and ATG4B, while reducing p62 accumulation and the p-ULK1/ULK1 ratio. These coordinated findings suggest that WVOE alleviates HS-induced suppression of hepatic autophagy-related signals, potentially through restoration of ULK1-associated signaling and downstream autophagosome formation-related markers.

WVOE administration ameliorated HS-induced autophagy dysfunction. Plant essential oils are widely recognized for their antioxidant, anti-inflammatory, and tissue-protective activities, which may enhance antioxidant defenses and support physiological resilience in heat-stressed poultry [31,32,33]. Moreover, extracts from *Clausena lansium* leaf extracts and leaf-derived bioactive constituents exhibit lipid-lowering and hepatoprotective activities [13,34]. Our previous GC-MS profiling revealed that WVOE is mainly composed of aromatic hydrocarbons, terpenoids and their oxygenated derivatives, with various bioactive constituents identified, including 5,5-dimethyl-1-vinylbicyclohexane, palmitic acid, β-ocimene, phytol, α-bisabolol, and caryophyllene oxide [14]. Among these constituents, α-bisabolol and caryophyllene oxide have been reported to possess antioxidant, anti-inflammatory, hepatoprotective, and autophagy-modulating activities [35,36]. Moreover, our previous work demonstrated that WVOE exerted anti-infective effects against Staphylococcus aureus by regulating the autophagy pathway [12]. Network pharmacology and molecular docking analysis further suggested that WVOE mitigate HS-induced injury in poultry through the modulation of apoptosis, autophagy, and NOD-like receptor signaling pathways [37]. Consistent with this prediction, the present study showed that WVOE alleviated several indices of HS-associated hepatic injury, accompanied by changes in autophagy-related marker expression and signaling.

Autophagy and ferroptosis are closely interconnected and mutually regulated processes, that contribute to the initiation and progression of various stress-related injuries [38,39]. Ferritinophagy may increase ferroptosis susceptibility by promoting intracellular iron release, whereas impaired autophagic function can also facilitate ferroptosis [40]. Zhang et al. reported that autophagy impairment caused SQSTM1/p62 accumulation, which in turn promotes ACSL4-mediated ferroptosis. Similarly, blockage of autophagy leads to the massive accumulation of SQSTM1/p62, an autophagy adaptor protein, which subsequently promotes ferroptosis through the exacerbation of oxidative stress and lipid peroxidation [41]. In a diabetic liver injury model, autophagy was also shown to regulate ferroptosis progression by regulating ACSL4 degradation [42]. Palmitic acid, an active constituent of WVOE, has been reported to modulate ferroptosis-related processes by increasing lipid peroxidation, MDA, and Fe^2+^ levels, and altering ferroptosis-related markers expression, including ACSL4, SLC7A11, and GPX4 [43]. Ferroptosis is characterized by impaired antioxidant defense and excessive lipid peroxidation. GPX4 is a central hub in preventing ferroptosis by converting toxic lipid hydroperoxides into non-toxic lipid alcohols, a process that strictly requires GSH as an essential co-substrate [44]. In the present study, HS decreased hepatic GSH levels and GPX4/SLC7A11 expression while increasing MDA content and ACSL4 expression. These findings suggest that HS weakened hepatic anti-lipid peroxidation defenses and induced ferroptosis-related molecular alterations. Supplementation with WVOE or VitC partially reversed these alterations, as reflected by increased GSH levels, reduced MDA content, upregulated GPX4 and SLC7A11 expression, and decreased ACSL4 expression. These findings indicate that WVOE may alleviate HS-induced lipid peroxidation by improving hepatic antioxidant defense.

HSP70 is a classical heat-responsive chaperone that is rapidly induced under thermal stress and contributes to cellular adaptation by maintaining protein homeostasis and limiting stress-induced damage [45]. Previous studies have shown that heat stress increases HSP70 expression in poultry tissues, including the liver, and this response is generally considered an adaptive defense mechanism against thermal and oxidative injury [46]. However, persistent or excessive HSP70 induction may also reflect sustained cellular stress and tissue injury. Consistent with previous studies in poultry [22], HS significantly increased hepatic HSP70 protein expression together with IL-1β and IL-6, indicating activation of stress and inflammatory responses. Both WVOE treatments reduced HSP70 protein expression and decreased HSP70 and IL-1β mRNA expression, while WVOE-L also reduced IL-6 protein expression. These findings suggest that WVOE may attenuate HS-induced hepatic stress and inflammation, thereby contributing to the improvement of liver injury-related indices observed in this study.

Several limitations should be acknowledged. First, WVOE was administered by gavage to ensure accurate dosage and reduce individual variation in intake, but this route is impractical under commercial broiler production conditions. Its practical application should therefore be further evaluated through drinking-water or feed supplementation. Second, although Tween 80 is widely used as an emulsifier, no vehicle control containing Tween 80 alone was included in the present study. Therefore, the potential contribution of the emulsifier cannot be completely excluded. Third, WVOE-H and WVOE-L did not show a consistent linear dose–response relationship. Previous studies have shown that phytochemicals and natural bioactive antioxidants may exhibit biphasic, threshold-dependent, or non-linear dose responses in complex biological systems [47,48]. This non-linear response of WVOE may be related to the complex chemical composition. The protective effects of WVOE may not simply increase with dose but be influenced by synergistic interactions among multiple active constituents and non-linear biological responses. Because only two doses were tested, the optimal effective dose could not be fully determined. Future studies should include additional dose levels and further assess the optimal dosage, tolerance, toxicological safety, and production benefits of WVOE under commercial broiler feeding conditions.

## 5. Conclusions

In conclusion, WVOE alleviated HS-associated liver injury, while WVOE-L improved feed efficiency in broilers, accompanied by modulation of mTOR/ULK1-related autophagy and ferroptosis-related antioxidant and lipid peroxidation pathways. WVOE may provide added value for wampee leaf utilization and reduce resource waste, but its practical application through drinking water or feed under commercial conditions requires further evaluation.

## Figures and Tables

**Figure 1:**
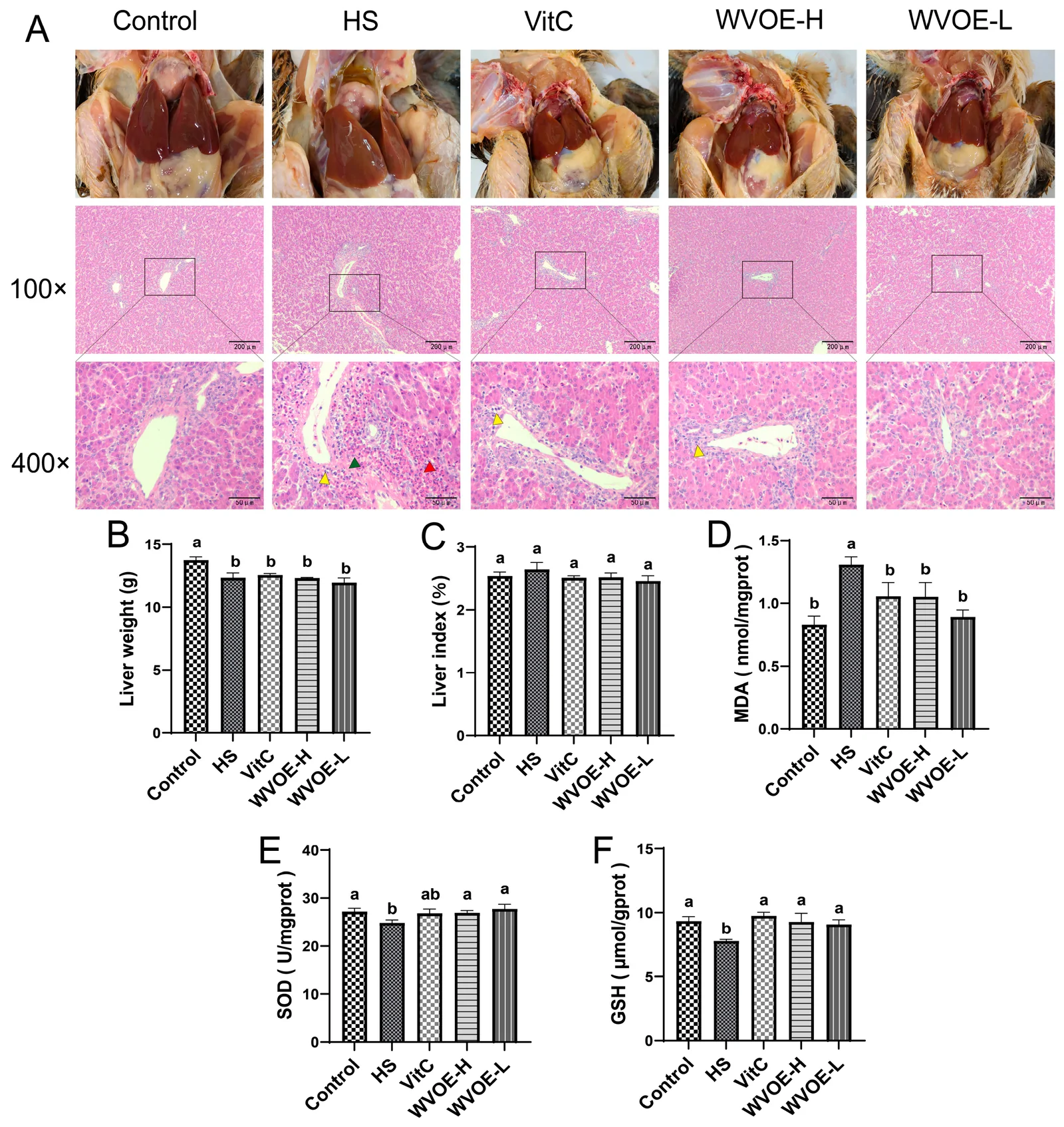
Effects of WVOE on hepatic changes in broilers under heat stress. (**A**) Liver pathological changes, including necropsy changes and histopathological changes. Yellow triangle represents inflammatory cell infiltration. Red triangle represents periportal erythrocyte extravasation. Green triangle represents hepatocellular necrosis. Representative gross and H&E-stained liver observations were selected to reflect the typical features of each treatment group. (**B**,**C**) Liver weight and liver index of broilers (24 broilers per group, 8 broilers per replicate cage). (**D**–**F**) Hepatic oxidative stress indices (12 liver samples per group, 4 broilers per replicate cage). Cage means were used as the experimental units (*n* = 3 replicate cages per group). Different superscript letters indicate significant differences among groups (*p* < 0.05).

**Figure 2:**
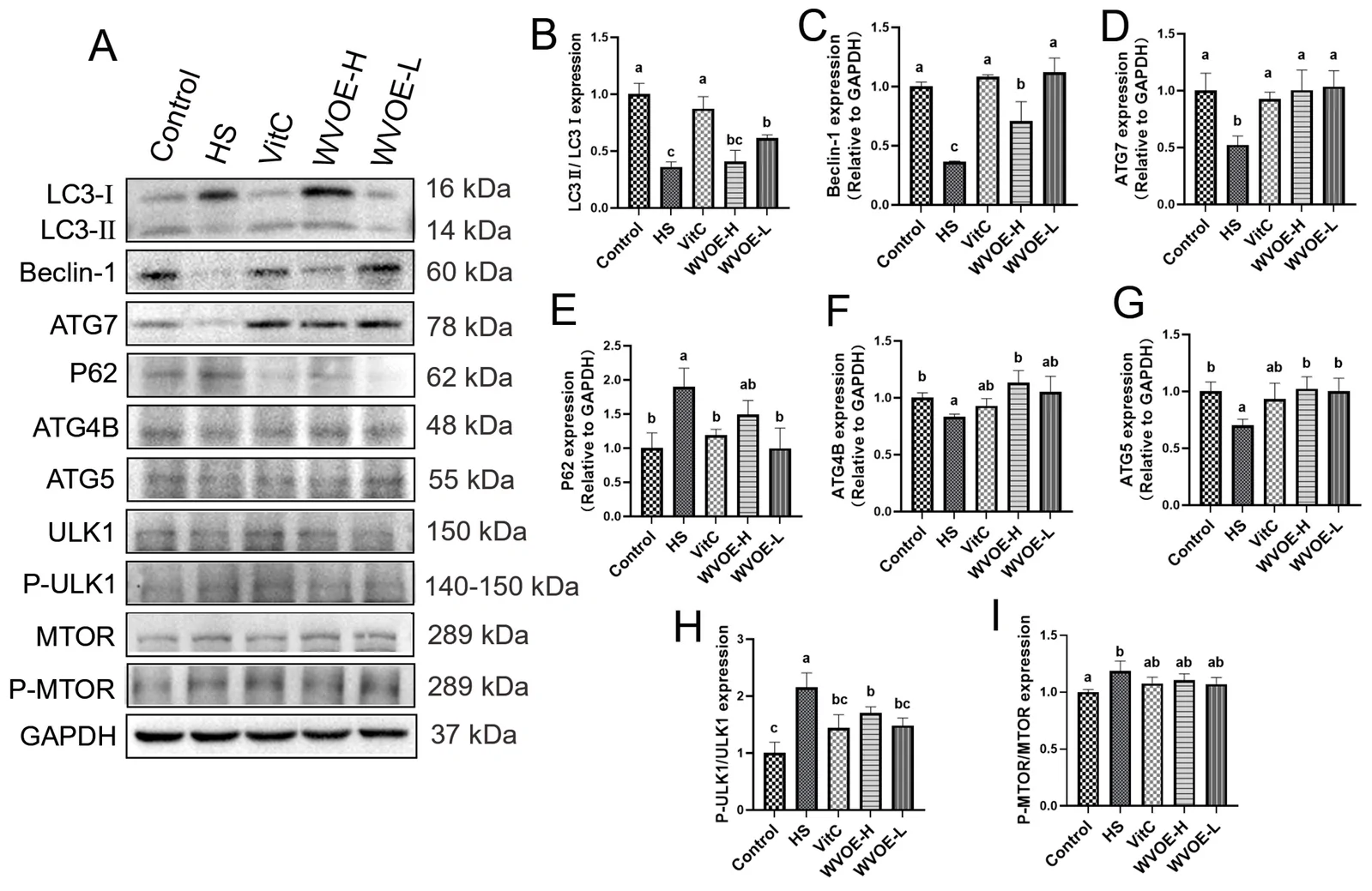
Effects of WVOE on autophagy-related protein expression in broilers under heat stress. (**A**) Proteins expressions in liver of broilers were measured by Western blotting, and (**B**–**I**) the relative protein expressions were detected. Three liver samples per group were analyzed, with one broiler selected from each replicate cage (*n* = 3 replicate cages per group). Different superscript letters indicate significant differences among groups (*p* < 0.05).

**Figure 3:**
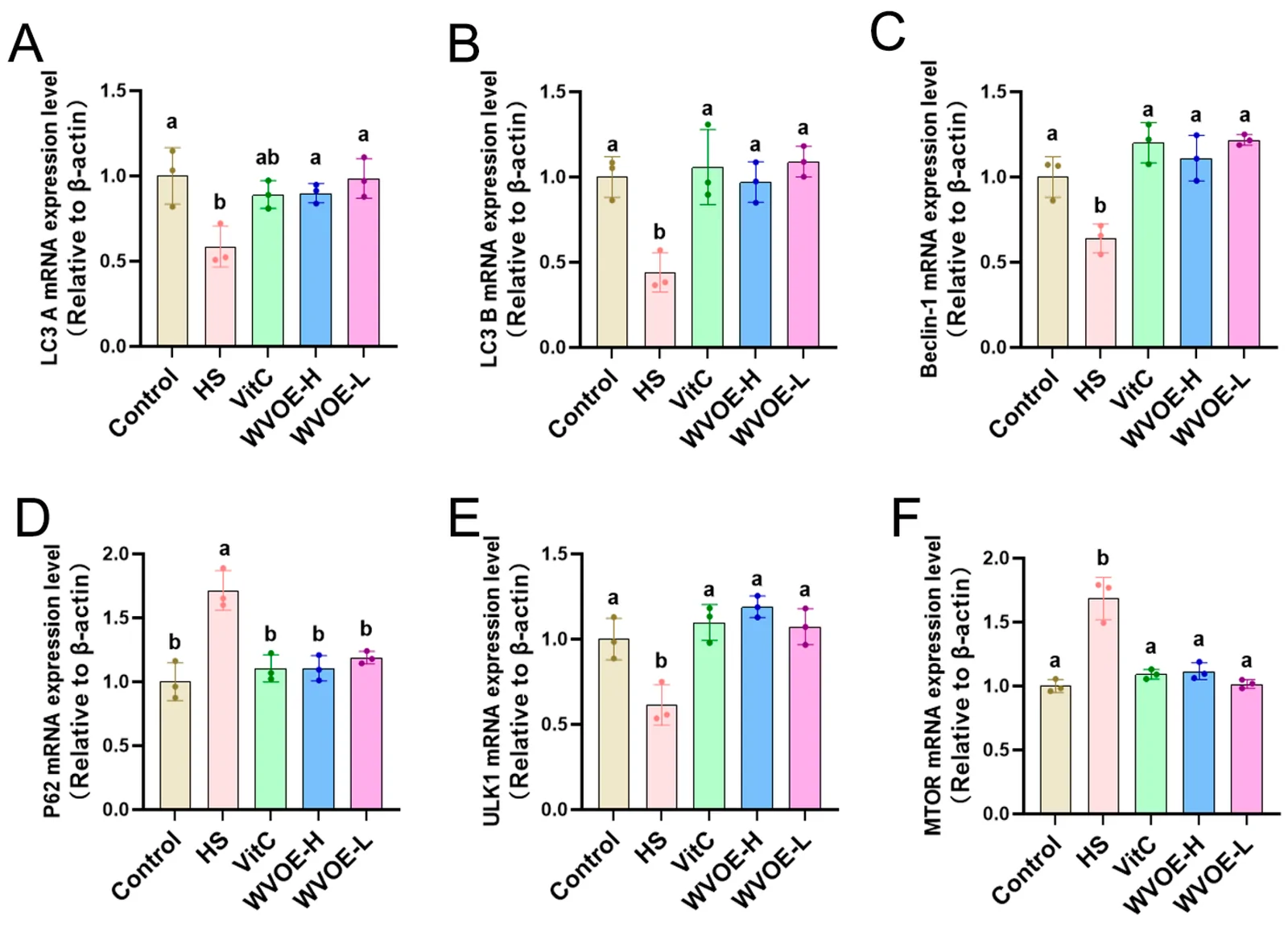
Effects of WVOE on the mRNA expression levels of autophagy-related genes in broilers under heat stress. (**A**) LC3A, (**B**) LC3B, (**C**) Beclin-1, (**D**) P62, (**E**) ULK1 and (**F**) MTOR gene expressions in liver of broilers were measured by RT-PCR. Six liver samples per group were analyzed, with two broilers selected from each replicate cage. Cage means were used as the experimental units (*n* = 3 replicate cages per group). Different superscript letters indicate significant differences among groups (*p* < 0.05).

**Figure 4:**
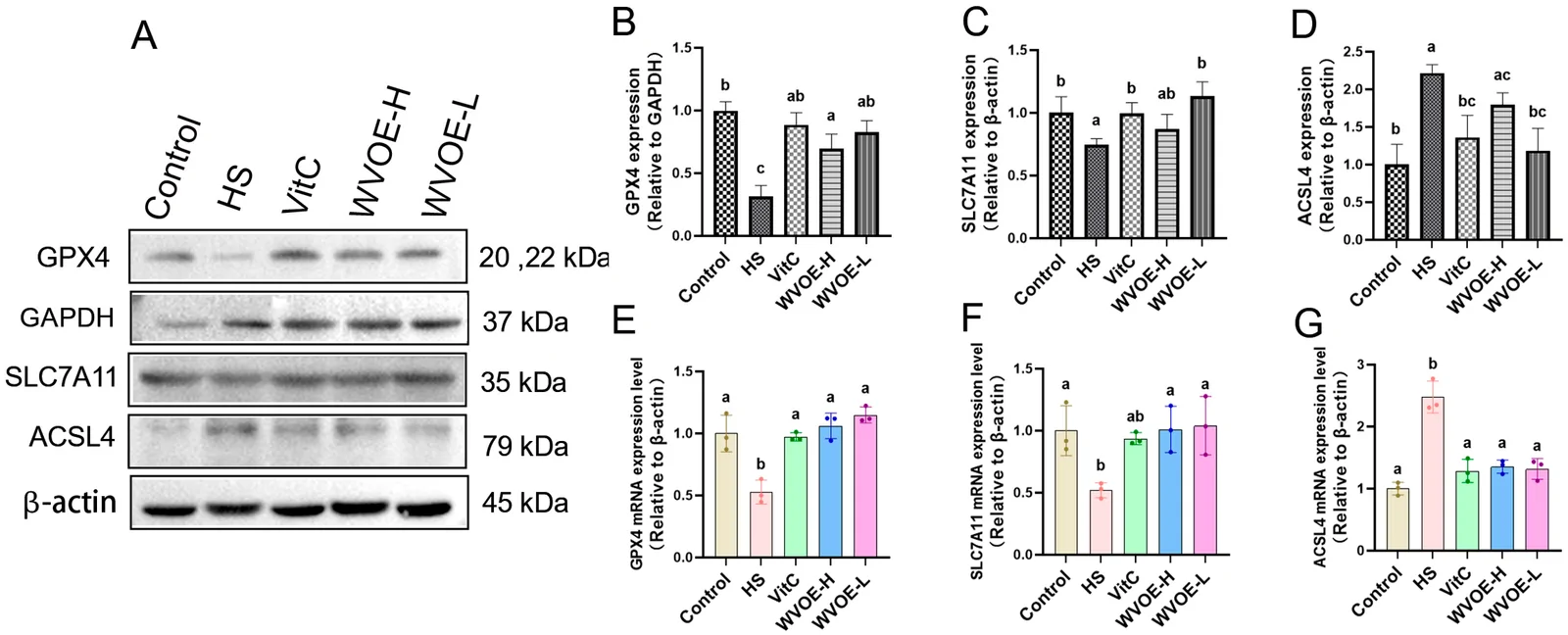
Effects of WVOE on ferroptosis-related markers in broilers under heat stress. (**A**–**D**): Hepatic protein expressions measured by Western blotting using three liver samples per group, with one broiler selected from each replicate cage. (**E**–**G**): Hepatic gene expression measured by RT-PCR using six liver samples per group, with two broilers selected from each replicate cage. Cage means were used as the experimental units (*n* = 3 replicate cages per group). Different superscript letters indicate significant differences among groups (*p* < 0.05).

**Figure 5:**
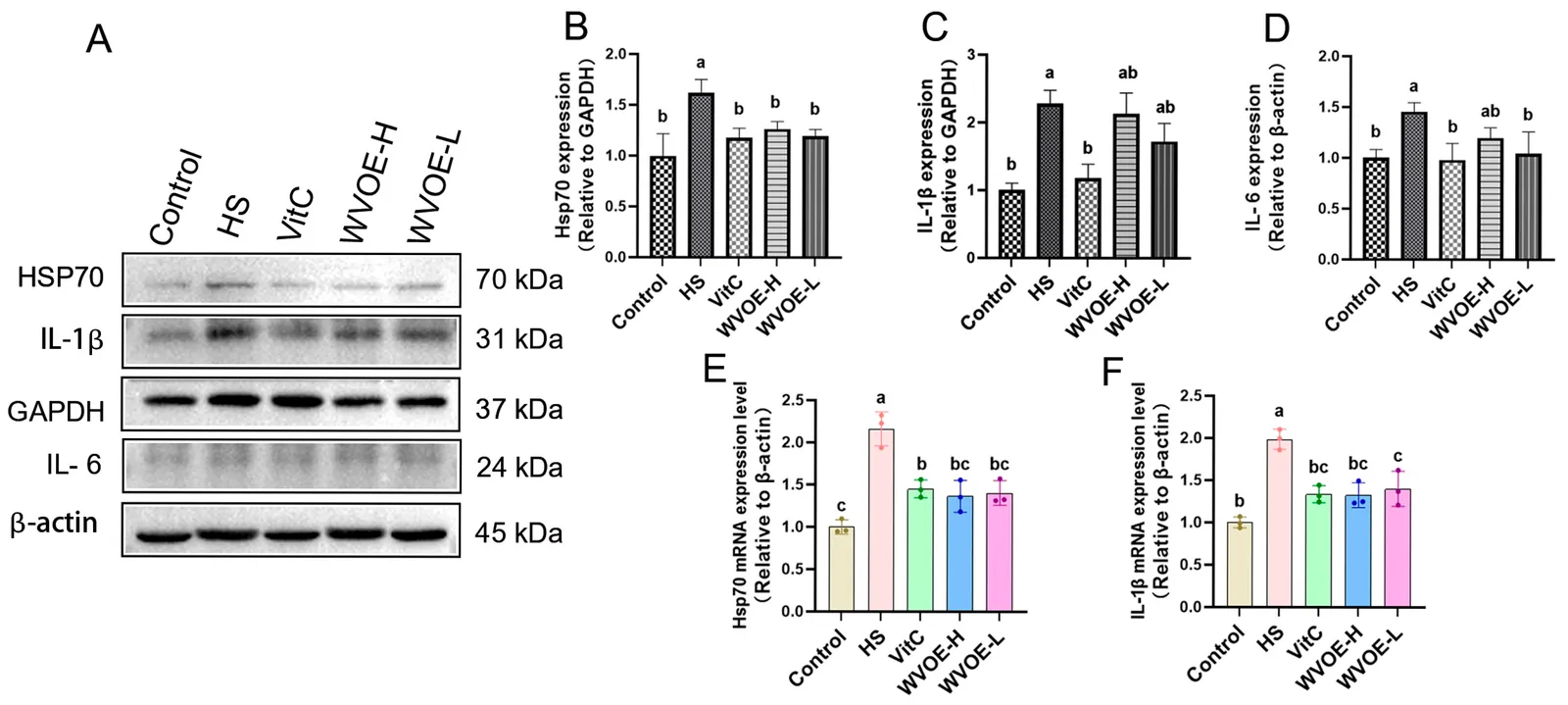
Effects of WVOE on inflammation-related molecular markers in broilers under heat stress. (**A**–**D**): Hepatic protein expression measured by Western blotting using three liver samples per group, with one broiler selected from each replicate cage. (**E**,**F**): Hepatic gene expression determined by qRT-PCR using six liver samples per group, with two broilers selected from each replicate cage. Cage means were used as the experimental units (*n* = 3 replicate cages per group). Different superscript letters indicate significant differences among groups (*p* < 0.05).

**Table 2:** Primer sequences for qRT-PCR.

Gene	Accession Number	Amplicon Size (bp)	Forward Primer (5′–3′)	Reverse Primer (5′–3′)
mTOR	XM_417614.8	103	AGCCAGGATTCTTCGGACTACAG	TCATCAGTGCCACCATAGAGACAG
ULK1	XM_415091.8	104	CCTTCACACTATGCGGACACTTAG	CCCGATGGATGATGCCTTTGC
Beclin-1	NM_001006332.1	97	CGTATGGCAACCACTGCTATT	TTATTGTCCCAGAAGAACCTCAG
LC3A	XM_040688401.2	143	TTACACCCATATCAGATTCTTG	ATTCCAACCTGTCCCTCA
LC3B	NM_001031461.2	193	ATTCCAACCTGTCCCTCA	AGTGAAGTGTAGCAGGATGA
HSP70	NM_001006685.2	112	GGTAAGCACAAGCGTGACAATGCT	TCAATCTCAATGCTGGCTTGCGTG
p62	XM_040682727.2	240	GACCCAGCCAAGACTACCAT	CAGAGGCATGTAGTTTCGGC
IL-1β	NM_204524.2	140	CACGGCAGCACTGGATAATGACC	CACTGGGCATCAAGGGCTACAAG
GPX4	NM_204220.3	153	TCGGCCACCTCCATCTACGAC	GTGCAGATCGACGAGCTGAGTG
SLC7A11	XM_040670528.2	90	GCTGTCGTGACGGTGCCTAATG	CTTCTCTTGTGGCTGCCTGCTG
ACSL4	XM_046917348.1	142	ACCGTTGTTATCTTCTGCGAGACC	ACGCTCCACATTCATTGAGACCATAG
β-actin	NM_205518.2	289	ACGTCTCACTGGATTTCGAGCAGG	TGCATCCTGTCAGCAATGCCAG

**Table 3:** Effect of WVOE on growth performance of broilers under heat stress.

Group	Initial Body Weight (g)	Final Body Weight (g)	ADG (g)	ADFI (g)	FCR
Control	305.25 ± 2.18 ^a^	542.59 ± 14.29 ^c^	33.91 ± 1.92 ^a^	52.73 ± 3.80 ^a^	1.56 ± 0.19 ^b^
HS	301.47 ± 1.87 ^a^	468.41 ± 6.78 ^b^	23.85 ± 0.12 ^c^	46.89 ± 3.54 ^ab^	1.97 ± 0.16 ^a^
VitC	301.69 ± 4.38 ^a^	500.31 ± 11.28 ^a^	28.37 ± 1.12 ^b^	49.57 ± 2.16 ^ab^	1.75 ± 0.02 ^ab^
WVOE-H	302.07 ± 5.88 ^a^	490.17 ± 12.61 ^ab^	26.87 ± 1.27 ^bc^	46.05 ± 2.88 ^ab^	1.71 ± 0.03 ^ab^
WVOE-L	303.45 ± 1.77 ^a^	486.19 ± 6.94 ^ab^	26.1 ± 0.19 ^bc^	43.23 ± 0.86 ^b^	1.66 ± 0.04 ^b^

Note: Cage means were used as the experimental units for initial body weight, final body weight, average daily feed intake (ADFI), average daily gain (ADG), and feed conversion ratio (FCR) (*n* = 3 replicate cages per group, 8 broilers per cage). Different superscript letters within the same column indicate significant differences among groups (*p* < 0.05).

**Table 4:** Serum biochemical analysis of broilers under heat stress.

Group	TP (g/L)	ALT (U/L)	AST (U/L)	ALB (g/L)	LDH (U/L)	T-Bil-V (µmol/L)	D-Bil-V (µmol/L)
Control	32.11 ± 0.70 ^a^	1.91 ± 0.10 ^c^	215.83 ± 4.42 ^b^	13.08 ± 0.51 ^a^	55.36 ± 0.91 ^b^	34.16 ± 0.42 ^a^	1.12 ± 0.03 ^a^
HS	26.34 ± 2.01 ^c^	3.00 ± 0.12 ^a^	274.45 ± 2.73 ^a^	9.68 ± 0.36 ^c^	65.58 ± 0.93 ^a^	32.21 ± 1.59 ^ab^	1.04 ± 0.01 ^a^
VitC	30.57 ± 0.88 ^ab^	2.06 ± 0.07 ^bc^	222.58 ± 3.75 ^bc^	12.14 ± 0.26 ^b^	56.75 ± 1.58 ^b^	34.08 ± 0.06 ^a^	1.08 ± 0.07 ^a^
WVOE-H	27.9 ± 0.65 ^bc^	2.76 ± 0.05 ^a^	227.13 ± 1.29 ^c^	11.99 ± 0.26 ^b^	59.38 ± 2.07 ^b^	33.56 ± 0.60 ^ab^	1.08 ± 0.05 ^a^
WVOE-L	28.53 ± 0.43 ^bc^	2.25 ± 0.12 ^b^	227.65 ± 4.08 ^c^	12.71 ± 0.15 ^ab^	57.00 ± 0.13 ^b^	31.33 ± 1.16 ^b^	1.09 ± 0.05 ^a^

Abbreviations: TP, total protein; ALT, alanine aminotransferase; AST, aspartate aminotransferase; ALB, albumin; LDH, lactate dehydrogenase; T-Bil, total bilirubin; D-Bil, direct bilirubin. Different lowercase letters within the same column indicate significant differences among groups (*p* < 0.05). For all serum biochemical parameters, cage means were used as the experimental units (*n* = 3 replicate cages per group, 8 broilers per cage).

## Data Availability

All data supporting the findings of this study are included in the article. No additional datasets were generated or analyzed.

## Abbreviations

The following abbreviations are used in this manuscript:

WVOE	*Clausena lansium* (Lour.) Skeels (wampee) leaves volatile oil emulsion
HS	heat stress
ADG	average daily gain
FCR	feed conversion ratio
ADFI	average daily feed intake
AST	aspartate aminotransferase
ALT	alanine aminotransferase
TP	total protein
ALB	albumin
MDA	malondialdehyde
SOD	superoxide dismutase
GSH	glutathione
T-Bil	total bilirubin
D-Bil	direct bilirubin
LDH	lactate dehydrogenase
